# Recent progress in sensing application of metal nanoarchitecture-enhanced fluorescence

**DOI:** 10.1039/d0na01050b

**Published:** 2021-03-09

**Authors:** Meiling Wang, Min Wang, Ganhong Zheng, Zhenxiang Dai, Yongqing Ma

**Affiliations:** Anhui Key Laboratory of Information Materials and Devices, School of Physics and Materials Science, Anhui University Hefei 230039 China yqma@ahu.edu.cn; Institute of Physical Science and Information Technology, Anhui University Hefei 230039 China

## Abstract

Fluorescence analytical methods, as real time and *in situ* analytical approaches to target analytes, can offer advantages of high sensitivity/selectivity, great versatility, non-invasive measurement and easy transmission over long distances. However, the conventional fluorescence assay still suffers from low specificity, insufficient sensitivity, poor reliability and false-positive responses. By exploiting various metal nanoarchitectures to manipulate fluorescence, both increased fluorescence quantum yield and improved photostability can be realized. This metal nanoarchitecture-enhanced fluorescence (MEF) phenomenon has been extensively studied and used in various sensors over the past years, which greatly improved their sensing performance. Thus in this review, we primarily give a general overview of MEF based sensors from mechanisms to state-of-the-art applications in environmental assays, biological/medical analysis and diagnosis areas. Finally, their pros and cons as well as further development directions are also discussed.

## Abstract 2

Studies and research advances of integrated plasmon metal nanoarchitectures in fluorescence sensing applications were reviewed.

## Graphical abstract

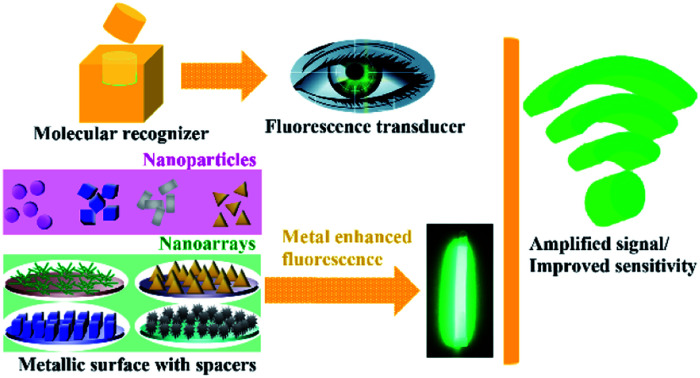

## 1 Introduction

Fluorescence-based techniques are simple and universal analytical methods extensively used in trace detection, biomedical imaging, diagnosis, optoelectronics and forensics.^1–7^ As a real time and *in situ* analytical approach to target analytes, fluorescence-based sensors can offer advantages of high sensitivity, high selectivity, non-invasive measurements and easy transmission over long distances.^7–9^ However, they still suffer from shortcomings, such as low fluorescence quantum efficiency, environment-dependent intensity and photobleaching, which severely hinder their wider application.

**Fig. 1:**
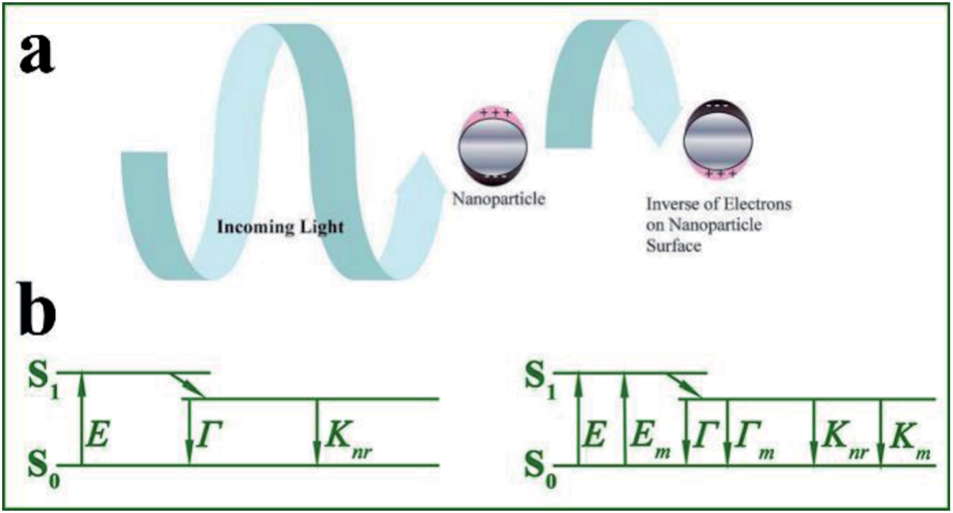
(a) Interactions between light and metal nanoparticles induce absorption and scattering of incident light. Schematic shows the induced surface charge oscillations by the external light field. Reprinted from ref. 1 with permission. (b) Jablonski diagram in the absence (left) and presence (right) of a metal nanostructure. *E* – excitation rate, *Γ* – radiation rate, *K*_nr_ – nonradiation decay rate, *E*_m_ – metal-enhanced excitation rate, and *Γ*_m_ – metal enhanced-radiation rate.

Localized surface plasmons (LSPs) refer to free electron collective oscillations induced by external electromagnetic waves, in confined metal nanostructures such as nanoparticles (see Fig. 1a). Resonance may occur when the light frequency matches that of the conduction electron oscillation. Under this condition, the electric field near the nanoparticle surface is largely enhanced and the metal optical extinction is greatly increased.^10,11^ Thus LSPs offer unique absorption and scattering properties to metallic nanoparticles.^12,13^ It should be noted that absorption (corresponding to nonradiative decays) induces fluorescence quenching of nearby fluorophores, while scattering contributes to fluorescence enhancement. And the proportion of scattering in total extinction spectra greatly affects the degree of fluorescence enhancement induced by these metallic nanoparticles. Silver and gold nanoparticles with larger sizes are preferably used for fluorescence enhancement, as for smaller ones, absorption loss tends to dominate.^14^

Based on the above theory, we summarized that metal nanoarchitecture-enhanced fluorescence (MEF, firstly reported in the 1960s^15,16^ and firstly used for biosensors in 1991) arises from resonant interactions between emission dipoles and LSPs of metal nanoarchitectures (see Fig. 1a). The enhanced electric field experienced by fluorophores gives rise to extra increased excitation rates *E*_m_, while the resultant coupling between fluorophores and nearby metal nanoparticles induces extra radiative rates *Γ*_m_. This process can be more easily understood using the Jablonski diagram shown in Fig. 1b. Thus MEF can effectively enhance the fluorescence quantum efficiency of fluorophores by increasing both excitation and radiative decay rates.^14,17–19^ To obtain effective MEF, there should be overlaps between excitation/emission spectra and the metal plasmon bands. It should be noted that, as a result of enhanced radiative decay rates, shortened effective fluorescence lifetimes can be obtained, which greatly improved the photostability of fluorophores.^2,14,20^ Thus MEF can solve the inherent shortcomings of fluorophores by improving both quantum efficiency and stability.

So far, LSPs have been used to enhance the emission of dyes,^17,19,21,22^ rare earth elements,^23–28^ quantum dots (QDs),^14,29–43^ carbon dots,^36,38^ carbon nanotubes,^44^ gold nanoclusters^45,46^ and even upconversion fluorophores.^33,47,48^ And this enhanced fluorescence has already been extensively applied in environmental analysis,^45,48–64^ tip-enhanced fluorescence spectroscopy,^65^ biotechnology^66^ (including fluorescence imaging,^67–71^ DNA mismatch investigation,^72,73^ immunoassays,^26,74–80^ bioassays,^28,38,42,81–100^ and investigation of biological mechanisms^67,101–103^), biomedical analysis (virus and bacteria detection^43,104^ and clinical diagnosis^105,106^) and so on. Characteristics and applications of MEF have been summarized in Table 1.

**Table 1:** Characteristics and applications of MEF

Characteristics of MEF	Enhanced fluorescence	Shortened fluorescence lifetimes	Improved fluorescence stability
Applications	Environmental analysis (including environmental pollutants such as organic pollutants and heavy metal ions)	Tip-enhanced fluorescence spectroscopy	Biotechnology and bioanalysis (including protein, DNA, ATP, enzymes and so on)

As reported, various kinds of metals have been used for MEF, such as gold,^21,22,34,48,49,75,100,107–113^ silver,^30,31,35,45,69,110,112,114–120^ aluminum,^121,122^ copper,^123–125^ nickel,^125,126^ chromium,^125,127^ zinc^125^ and so on. So far, MEF across the ultra-violet to the second near infrared wavelength range has been reported.^128^ Considering their visible and near infrared plasmon transitions, low damping associated with inter- and intra-band transitions,^129^ as well as easy fabrication and functionalization, gold and silver are most commonly used for MEF in practical applications.^130^ Gold and silver nanoparticles show plasmon frequencies decreasing with increased particle size, and thus their plasmon bands can be tuned over the entire visible and near infrared spectrum. Besides composition and size, plasmon transition frequencies can also be affected by shape and local dielectric environments of metallic nanostructures. Silver shows a much higher scattering efficiency than gold in the wavelength range of *λ* < 600 nm,^129,131^ while gold is known for its better biocompatibility and chemical stability.

MEF induced fluorescence intensity is principally determined by the following two factors: the degree of spectral overlap between fluorophore excitation and plasmon transitions, and the distance between the fluorophore and the metal surface.^14,132,133^ For fluorophores in close proximity (less than 5 nm) or even directly attached to the metal surface, quenching may occur as a result of the excited electron transfer to the metal *via* nonradiative decay ways.^134^ However, as the enhanced electric field decays nearly exponentially with distance, the fluorophore should not be too far from the plasmonic nanostructure. The efficiency of plasmon induced MEF is highest when they are separated by a few nanometers,^14^ and thus distance control is important.

To achieve a superior MEF effect, a core/shell structure or a sandwich structure is commonly used, which consists of a metal core/film, a dielectric shell/film as a spacer layer and a fluorophore layer.^10,135^ The spacer layer is usually a dielectric layer with precisely/atomically controlled thickness, and used to separate and connect the metal core/film and the fluorophore layer.^26,50,56,58,74,136^ Both the defect-free quality and the dielectric properties of the spacer can affect the plasmonic enhancement.^137^ In addition, an ideal spacer layer should play roles in protecting the metal from oxidation. Based on these theories, various spacer layers such as silica,^18,45,138,139^ alumina,^140^ polymers,^81,141,142^ graphene,^48,143,144^ biomolecules including aptamers and antibodies,^75,77,145–147^ BN,^17,19,148,149^ MoS_2_,^19^ WS_2_,^19^ natural halloysite nanotubes,^25^ carbon dots^144^ and carbon nanotubes^144^ have been reported. It should be pointed out that each kind of spacer layer has its advantages and disadvantages,^17,136^ and we can choose the most suitable one according to requirements.

**Fig. 2:**
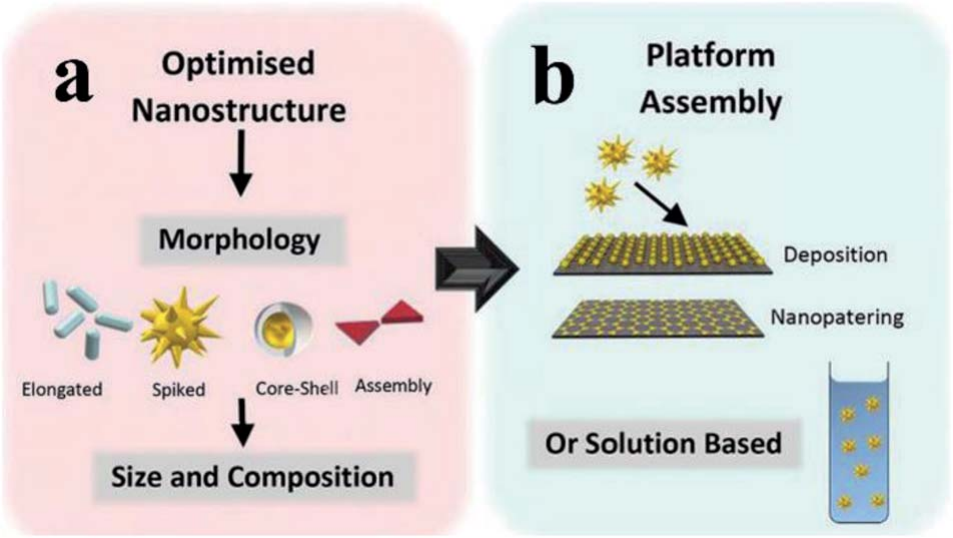
Schematic showing (a) the optimization of metal geometric nanostructures, including morphology, composition and size selection, and (b) integrated plasmon metallic nanostructure assembly (including chip film and solution-based platforms). Reproduced from ref. 128 with permission from the Royal Society of Chemistry.

To produce the integrated core/shell or sandwich plasmonic nanostructure, commonly the following three processes should be considered: preparation of the metallic nanostructure, coating of the spacer layer and immobilization of the fluorophore layer.^27,59–64,147^ Various geometrical nanostructures for MEF have been studied so far, and they can be categorized into two main groups: solution-based colloidal nanoparticles (NPs)^27,31,48,52,54,92,145,150^ and periodical/non-periodical plasmonic substrate chips,^45,51,64,74,108,135,151,152^ see Fig. 2. Design, preparation and performance study of these different geometrical plasmonic nanostructures are well summarized in previous reviews.^10,31,108,112,125,136,139,153^

In this review, after elaborating the related MEF theory and platform assembling method, in the following sections, we will review research advances and the implications/limitations of these integrated MEF plasmon nanostructures in fluorescence sensing applications. Finally, we will present our views on future directions and further perspectives in this area. We hope that this review can provide a comprehensive coverage of current development achievements in this field and put forward new perspectives for the future development of improved sensing performance.

## 2 MEF-based fluorescence sensing for environmental analysis

### 2.1 Detection of heavy metal ions

So far, most of the MEF sensing is performed using solution-based colloidal NPs, where plasmonic NPs are dispersed into analyte-containing solutions, as they are attractive for biological detection, bioimaging and nanophotonic applications.^136^

Among various types of spacer layers, silica is highly regarded for its ease of preparation, thickness controllability, light transparency, stability and biocompatibility.^154^ So far, such silica-coated core–shell NPs have been taken as the most successful class of hybrid plasmonic colloids. Various functions of silica shells are summarized in ref. 139. In this section, we will give a detailed introduction to Ag/Au@SiO_2_ core–shell colloidal NPs used in environmental analysis areas.

Heavy metal ions, such as Cu^2+^, Hg^2+^ and Pb^+^, are considered as persistent toxic pollutants, which mainly come from uncontrolled battery manufacturing, metal melting, automobile exhaust and old ship demolition.^155^ Once discharged into the environment, these heavy metals show high biotoxicity, degradation-resistance and bioaccumulation. Thus their trace detection in aqueous solutions is urgent and important.

Therefore, MEF-based fluorescence sensors for these heavy metal ions have been developed in virtue of their improved emission properties (see Table 2). For example, Kim and his co-workers fabricated a new kind of Ag@SiO_2_ core–shell NP, with silica chosen as the rigid spacer to adjust the distance between the Ag core and fluorophores, and Au_25_ nanoclusters modified on its surface acting as the fluorescence indicator.^45^ MEF of Au_25_ nanoclusters was studied with varied core sizes, shell thicknesses and excitations. And an enhancement factor of 7.4 was obtained under optimal conditions. Furthermore, the Au_25_-adsorbed Ag@SiO_2_ NPs were used for highly sensitive and selective ‘turn-off’ sensing of Cu^2+^, and it was proved that the turn-off ratio is 3.3 times larger than that of free Au_25_ nanoclusters under optimal conditions, indicating their much superior sensing ability for Cu^2+^ in aqueous solutions.

**Table 2:** MEF-based sensors for heavy metal ions

MEF sensors	Fluorophores used	Fluorescence enhancement factor	Target analytes	Relative merits (LOD refers to the limit of detection)
Ag@SiO_2_ core–shell NPs^58^	HPTS	4 and 9 fold with excitation of 405 and 455 nm respectively	pH	Ratiometric sensing; pH detection range of 5–9 was realized
Ag@SiO_2_ core–shell NPs^50^	2-AA	6.4	2-AA	Outstanding selectivity over co-existing polycyclic aromatic hydrocarbons
Ag@SiO_2_ (ref. 63)	Tetracycline	6.8	Tetracycline	LOD of 25 pM; real water sample analysis was realized
Ag/SiO_2_/SiO_2_ core–shell NPs and nanorods (NRs)^52^	FITC	2.63 and 3.5	Fe^3+^	LOD of 19.4 and 0.83 nM respectively; a prototype arduino based electronic device was fabricated
Ag NP based molecular beacons^147^	FAM	5.6	Hg^2+^	LOD of 1 nM; quantitative analysis in real lake water samples
MBs-aptamer/cDNA-Au@Ag15-GU^48^	Upconversion nanoparticles	4.5	Hg^2+^	Dual channel biosensor (SERS and FL); LOD of 0.33 and 1 ppb respectively; quantify Hg^2+^ in spiked tap water and milk samples; reproducibility, selectivity and anti-interfering ability
CSN-RhD^55^	Rhodamine derivatives	Not mentioned	Hg^2+^	Bimodal sensor (SERS and FL); with a linear detection range from 0.001 to 100 ppm and 0.01–100 ppm, and LODs of 0.94 and 5.16 ppb for MEF and SERS modes
ZnFe_2_O_4_@Au–Ag core–shell nanocomposite conjugated with double-stranded DNA^158^	Cy3	Not mentioned	Pb^2+^	Ratiometric fluorescence analysis in the range of 10^−12^ to 3 × 10^−6^ M; LOD of 3 × 10^−13^ M; good recyclability and selectivity; real-time visual detection

**Fig. 3:**
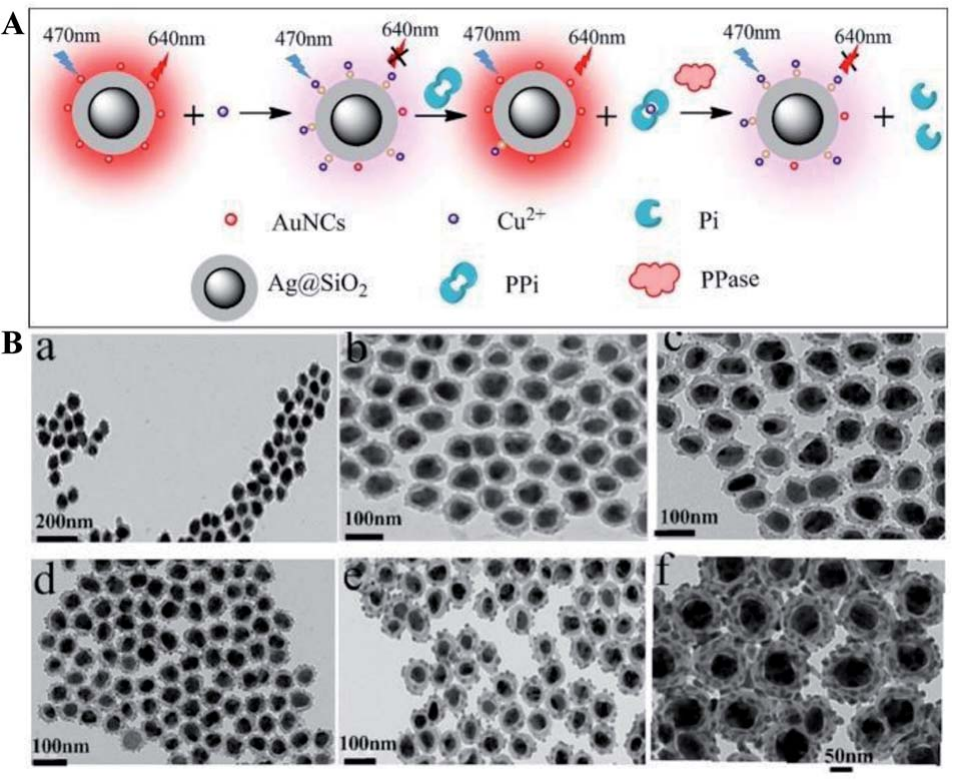
(A) Schematic of sensing mechanisms of multiple components with Ag@SiO_2_–AuNCs, AuNCs refer to Au nanoclusters and (B) TEM micrographs of composite Ag@SiO_2_ nanoparticles with different SiO_2_ spacer thicknesses: (a) ∼7 nm, (b) ∼10 nm, (c) ∼12 nm, (d) ∼15 nm, (e) ∼20 nm, and (f) ∼25 nm. Reprinted from ref. 56 with permission.

Ag@SiO_2_ based core–shell colloidal NPs of Ag@SiO_2_–AuNCs (AuNCs refer to Au nanoclusters) with good water dispersibility, high stability and good biocompatibility have been synthesized by Xu and his co-workers for multi-component detection of Cu^2+^, pyrophosphate (PPi) and pyrophosphatase (PPase) (Fig. 3). Interactions between silver cores and the outer Au NPs greatly enhanced the emission of Au NPs *via* improving their excitation efficiency, and the composite core–shell nanostructures were used for developing a sensing platform based on OFF–ON–OFF switching of the fluorescence signal in the presence of Cu^2+^, PPi and PPase, and the detection limits were 39 nM, 78.7 nM and 0.976 mU respectively. These sensing nanostructures have also been applied for fluorescence cellular imaging.^56^ However, unfortunately, their fluorescence imaging results can only be used for qualitative research of Cu^2+^, PPi or PPase, but not for quantitative research.

**Fig. 4:**
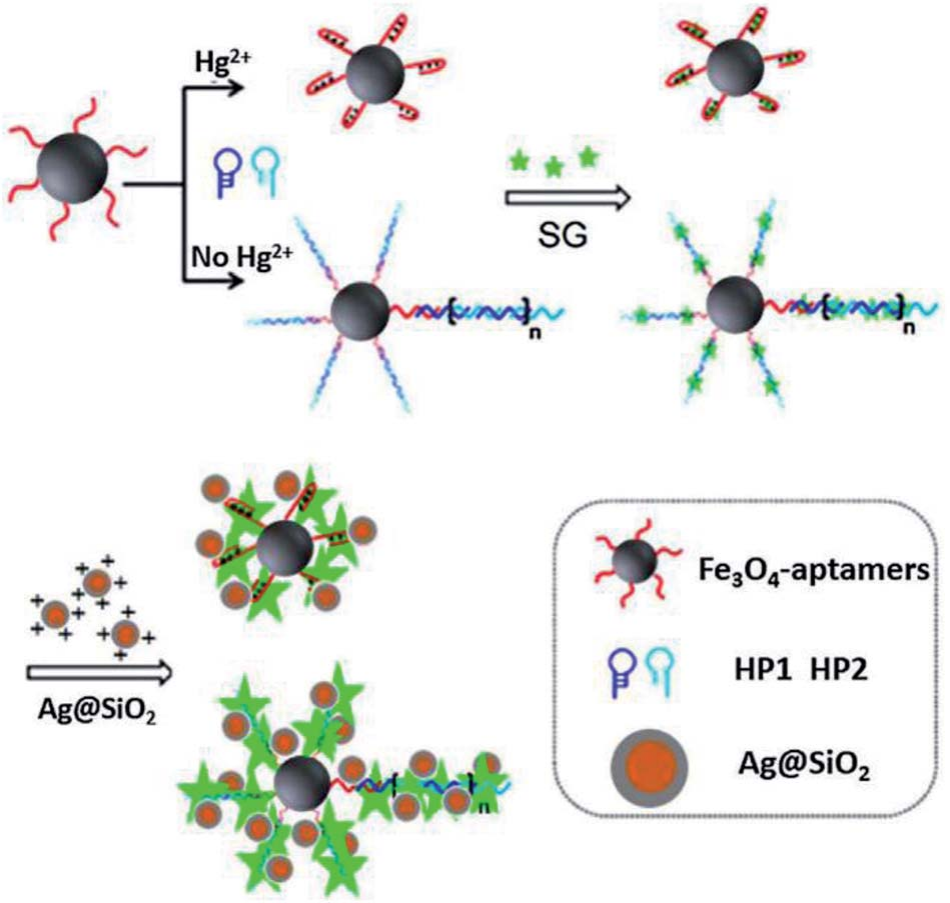
Schematic illustration of the sensing system for Hg^2+^ detection. Reprinted from ref. 63 with permission.

Sui and his co-workers reported an Ag@SiO_2_ based core–shell nanoprobe for Hg^2+^, using a sensing strategy by combining MEF and hybridization chain reaction (HCR). As shown in Fig. 4, in the absence of Hg^2+^, HCR occurred, resulting in the formation of long DNA chains with fluorescent indicator SYBR Green intercalated into the double DNA helix. Thereafter, positively charged Ag@SiO_2_ NPs were added after a magnetic separation process from the test solution, which will be electrostatically adsorbed onto negatively charged DNA chains, just as ‘smart dust’ to enhance the fluorescence signal. In the presence of Hg^2+^, the fluorescence signal gradually decreased with [Hg^2+^], and a detection limit of 25 pM was obtained under current experimental conditions.^63^ And this probe has already been used for selective detection of Hg^2+^ in real water.

Moreover, Rajbongshi and his co-workers synthesized fluorescein isothiocyanate (FITC) modified Ag/SiO_2_/SiO_2_ core–shell NPs and nanorods (NRs), and fluorescence quenching detection of Fe^3+^ was achieved using these plasmon nanostructures of different geometries. Lower detection limits of 19.4 and 0.83 nM were obtained for NPs (highest fluorescence enhancement factor of 2.63) and NRs (highest fluorescence enhancement factor of 3.5) respectively.^52^

**Fig. 5:**
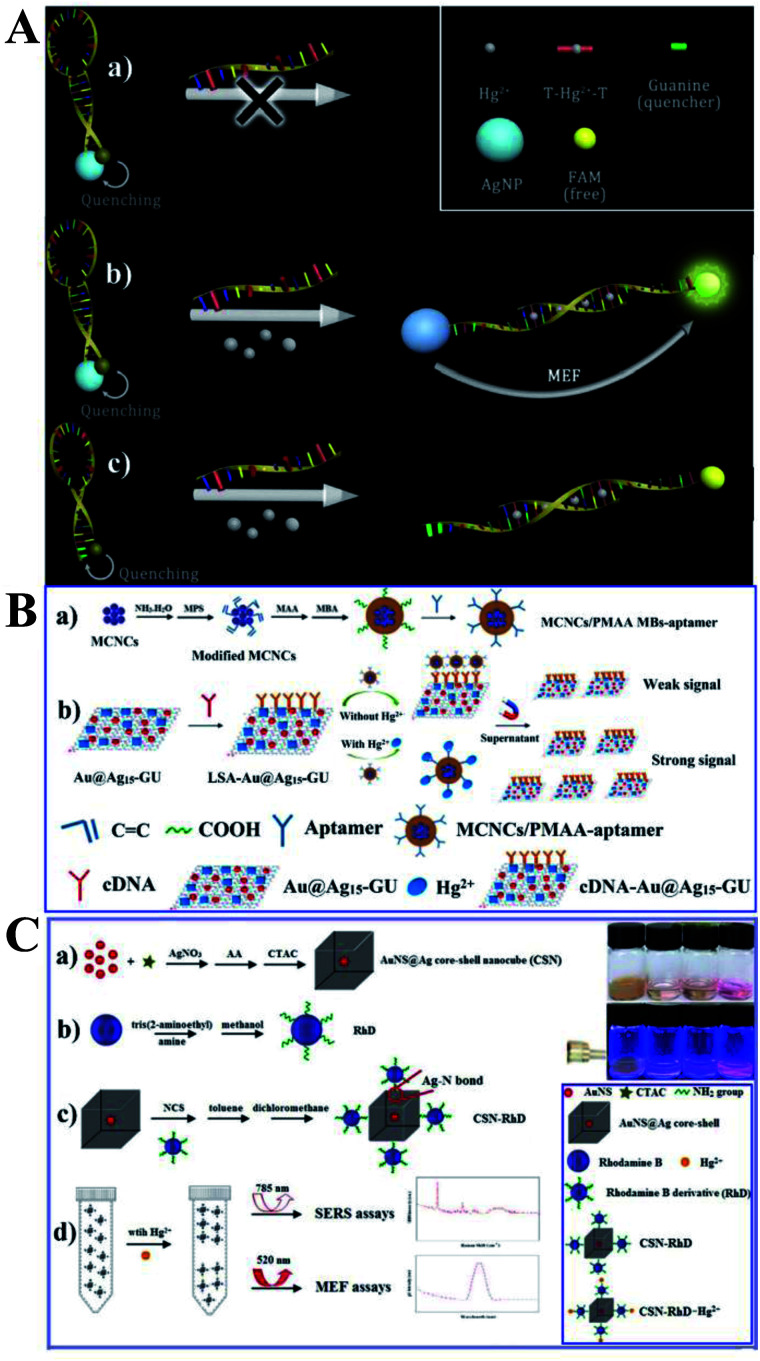
(A) Schematic showing the sensing principle of the proposed method, reprinted from ref. 147 with permission. (B) Schematic illustrations of the (a) fabrication and (b) sensing procedure of the dual-channel biosensor for the detection of Hg^2+^, reprinted from ref. 48 with permission. (C) Schematic illustration of the fabrication processes and sensing mechanisms of the proposed bimodal sensor for Hg^2+^, reprinted from ref. 55 with permission.

As shown in Fig. 5A, a new MEF-based probe was designed using the distance-dependent fluorescence quenching-enhancement effect. Hg^2+^ was detected *via* the formation of the thymine–mercuric–thymine structure, which can open the hairpin and induce distance changes between the fluorophore and the Ag NPs. This process can realize fluorescence dequenching and MEF induced by Ag NPs, and 1 nM Hg^2+^ was detected. In this work, Ag NPs were functionalized as both the quencher to reduce blank signals and the enhancement substrate for MEF, which greatly improved detection sensitivity. And this design principle can be universal for MEF-based probes.^147^

As Raman spectroscopy has attractive vibrational fingerprint features with a bandwidth of over 100 times smaller than fluorescence,^156^ by combining the high-level multiplexing and specificity of surface-enhanced Raman scattering (SERS) with the large area rapid read-out of fluorescence signal, the dual-mode optical analysis is emerging as a powerful sensing analytical tool especially in biological and biomedical applications.^156^ Furthermore, as reported, much more pronounced photostability can be obtained for fluorophores near to a mixed metal substrate than to a single one.^157^ Thus Li and her co-workers fabricated a dual-channel biosensor by immobilizing versatile signal indicator Au@Ag/graphene upconversion nanohybrids (Au@Ag–GU) onto the surface of magnetic beads (MBs) through the complementary pairing reaction between the aptamer and the complementary DNA (cDNA) (denoted as MBs-aptamer/cDNA-Au@Ag15-GU), with the conjugated aptamer used for specific capture Hg^2+^. And the obtained sensor can export dual channels of SERS and fluorescence signals for simultaneous Hg^2+^ detection. As shown in Fig. 5B, in the absence of Hg^2+^, the MBs-aptamer/cDNA-Au@Ag15-GU can be easily attracted to one side using an external magnet, and the resulting supernatant solution did not include Au@Ag15-GU, and thus no SERS or fluorescence signal was observed. However, in the presence of Hg^2+^, specific binding of the aptamer with Hg^2+^ occurred, resulted in liberation of some cDNA-Au@Ag15-GU into the supernatant, which revealed strong SERS and enhanced fluorescence signals with increasing [Hg^2+^]. The as-fabricated dual-channel biosensor showed excellent performances for Hg^2+^ with detection limits of 0.33 and 1 ppb for SERS and fluorescence mode respectively, under the optimized conditions. And it has also been used for quantify Hg^2+^ in spiked tap water and milk samples. SERS can be used to achieve accurate results while the fluorescence method gives a much wider linear range, cheaper instruments and good reproducibility. It should be noted that this strategy bridged the gap between fluorescence sensing and SERS assays, which broadens future applications of MEF-based sensing.

And on this basis, the same research group synthesized a novel bimodal sensor based on rhodamine derivative (RhD) grafted Au nanospheres@Ag core–shell nanocubes (denoted as CSN-RhD), which show both MEF and SERS dual signals for Hg^2+^ detection (see Fig. 5C). Both the SERS and MEF intensity increased with [Hg^2+^]. With an optimized Ag cubic shell thickness, this CSN-RhD showed wide linear ranges of 0.001–1000 ppm and 0.01–1000 ppm, and detection limits of 0.94 and 5.16 ppb for MEF and SERS mode, respectively. These excellent sensing performances for Hg^2+^ can be attributed to effective signal enhancement ability of the CSN plasmon nanostructures.^55^

**Fig. 6:**
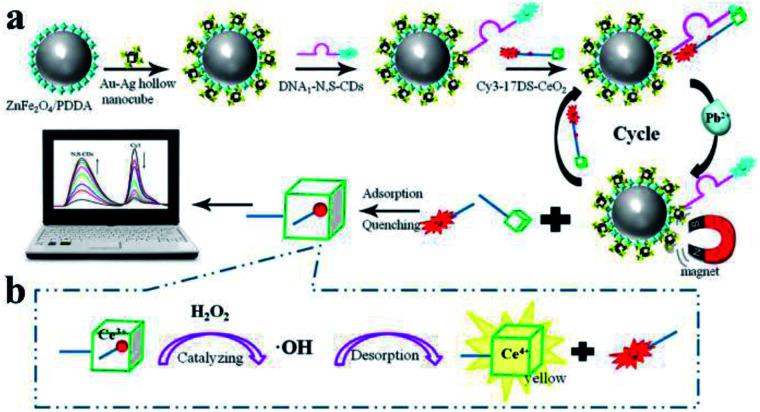
Schematic showing the fabrication and sensing processes of the Pb^2+^ biosensor. Reprinted Scheme 1 with permission from Linlin Liang, Feifei Lan, Shenguang Ge, Jinghua Yu, Na Ren and Mei Yan, *Anal. Chem.*, 2017, **89**, 3597–3605 (ref. 158). Copyright 2017 American Chemical Society.

Moreover, Liang and his co-workers developed another novel MEF ratiometric/naked eye bimodal biosensor for Pb^2+^, which was composed of a ZnFe_2_O_4_@Au–Ag core–shell bifunctional nanocomposite conjugated with double-stranded DNA (including the Pb^2+^-specific DNAzyme strand labeled with Cy3 and the corresponding substrate strand labeled with N,S-doped carbon dots (N,S-CDs)). The fluorescence of N,S-CDs was significantly quenched with the formation of double-stranded DNA, which brought N,S-CDs and the super quencher CeO_2_ into close proximity, see Fig. 6. At the same time, Cy3 fluorescence was enhanced by the MEF effect of Au–Ag core–shell hollow nanocubes. In the presence of Pb^2+^, the DNAzyme strand was activated, broken away from the substrate strand and cleaved at the cleavage site into two fragments (red dot in Fig. 6). This process resulted in dequenching of N,S-CDs emission, while the fluorescence of the Cy3 labeled fragment was efficiently quenched as it can be easily adsorbed onto the CeO_2_. As shown in Fig. 6b, the disengaged DNA/CeO_2_ complex could result in a color change after adding H_2_O_2_ as a result of CeO_2_ autocatalysis, and thus real-time visual detection of Pb^2+^ can be realized. At the same time, by using the good linear relationships between log(*I*_562_/*I*_424_) and log[Pb^2+^] in the range of 10^−12^ to 3 × 10^−6^ M, ratiometric fluorescence quantitative analysis of Pb^2+^ can be realized.^158^ Their work might provide potential applications for on-site and real-time Pb^2+^ detection in real water systems.

### 2.2 Analysis of pH and organic pollutants

Furthermore, MEF-based fluorescent sensors for pH and other organic pollutants were developed (as shown in Table 3). For example, a novel ratiometric sensor consisting of Ag@SiO_2_ core–shell NPs and a kind of pH sensitive dye of HPTS is developed by Bai and his co-workers for the pH assay.^58^ With a shell thickness of 8 nm, fluorescence was enhanced by 4 and 9 fold with excitation of 405 and 455 nm respectively. And the emission ratio of 513 nm excited by 455 nm to that excited by 405 nm *versus* pH in the range of 5–9 was determined, showing its potential application for pH detection in environmental and biological samples.

**Table 3:** MEF-based sensors for pH and organic pollutants

MEF sensors	Fluorophores used	Fluorescence enhancement factor	Target analytes	Relative merits (LOD refers to the limit of detection)
Ag@SiO_2_ core–shell NPs^58^	HPTS	4 and 9 fold with excitation of 405 and 455 nm respectively	pH	Ratiometric sensing; pH detection range of 5–9 was realized
Ag@SiO_2_ core–shell NPs^50^	2-AA	6.4	2-AA	With a wide linear range of 1–800 nM; outstanding selectivity over co-existing polycyclic aromatic hydrocarbons
Ag@HNTs-Cit-Eu^25^	Eu^3+^	Not mentioned	Tetracycline	LOD of 4.8 nM; visual detection
Hydrogel microarray entrapping QD-Ag@Silica and AChE^64^	QDs	Not mentioned	Paraoxon	LOD of 1.0 × 10^−10^ M; exhibiting sensitivities over three orders of magnitude higher than those without MEF effect

Trace detection of 2-aminoanthracene (2-AA), an aromatic amine, is of great significance for environmental monitoring. Recently, Jin and co-workers synthesized a kind of Ag@SiO_2_ core–shell NP with an ∼40 nm Ag core and an ∼7 nm SiO_2_ shell, which could efficiently increase 2-AA emission *via* MEF. Based on this theory, 2-AA was detected within a wide linear range of 1–800 nM, with outstanding selectivity over co-existing polycyclic aromatic hydrocarbons.^50^

Besides Ag/Au@SiO_2_ core–shell colloidal NPs, other MEF-based detection in environmental analysis using Ag/Au colloidal NPs with different spacers have been reported, which we will review in this section too.

**Fig. 7:**
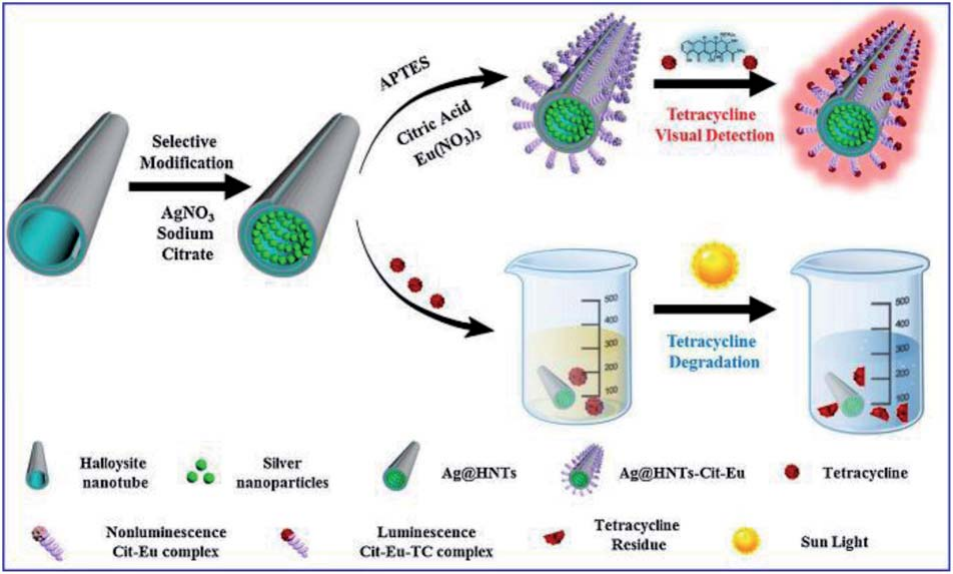
Schematical fabrication of the Ag@HNTs-Cit-Eu nanocomposite, and its schematic diagram for the detection and degradation of TC. Reprinted from ref. 25 with permission.

Tetracycline (TC), as a most frequently used antibiotic, also causes water pollution and enter the human body through the food chain, posing hazards to both the ecological environment and human health. Thus ultrasensitive detection of TC residues in water environments is necessary.^25^ Xu and his co-workers developed a smart silver-enhanced fluorescence platform *via* a simple modification of the interior and external surfaces of natural halloysite nanotubes (HNT) with Ag nanoparticles and Cit-Eu nanoprobes (schematically shown in Fig. 7). The appropriate thickness of the HNT walls results in effective MEF of Cit-Eu. And this Ag@HNTs-Cit-Eu nanocomposite was used for ultra-sensitive detection of TC with a detection limit of 4.8 nM.^25^

As compared to solution-based colloidal NPs, attaching NPs to a substrate to integrate a plasmonic nanochip can reduce the tedious washing steps, but usually very low and position-dependent enhancement factors are obtained using this method as a result of random NP distributions. Considering this flaw, researchers have explored various ordered nanostructure arrays with much better plasmonic coupling between adjacent nanostructures and more hot-spot generation in nanogaps.

**Fig. 8:**
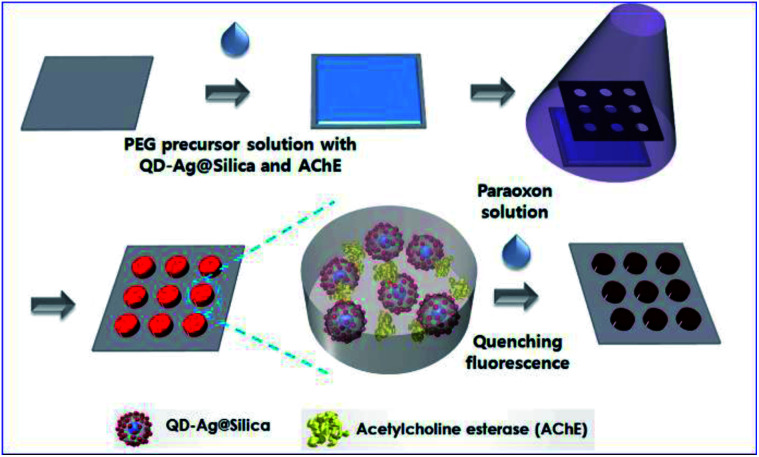
Schematic showing fabrication and sensing processes of the hydrogel microarray biosensor for paraoxon. Reproduced from ref. 64 with permission from the Royal Society of Chemistry.

For example, Kim and his co-workers developed an enzyme-based miniaturized biosensor of hydrogel microarray entrapping QD-Ag@Silica and AChE for detection of neurotoxic paraoxon. In this design, MEF of Ag NPs was adopted to improve sensing performance, and paraoxon was detected *via* amplified QD fluorescence quenching once exposed to *p*-nitrophenol produced by the AChE-catalyzed hydrolytic reaction (see Fig. 8).^64^ This biosensor exhibited sensitivities (1.0 × 10^−10^ M) over three orders of magnitude higher than those without MEF effect. Its successful integration with microfluidic systems further demonstrated potential applications for micro-total-analysis-systems.

## 3 MEF-based sensing for the bioassay

Today, to improve disease curative and survival rates, early diagnosis is crucially important. Very low concentrations of biomarkers must be detected with great efficiency and reliability at preliminary stages. As a result of improved optical properties of fluorophores, MEF allows for much lower detection limits and much earlier diagnosis. Thus great efforts have been made by researchers in developing MEF-based sensing for bioassays.

### 3.1 Immunoassay

**Fig. 9:**
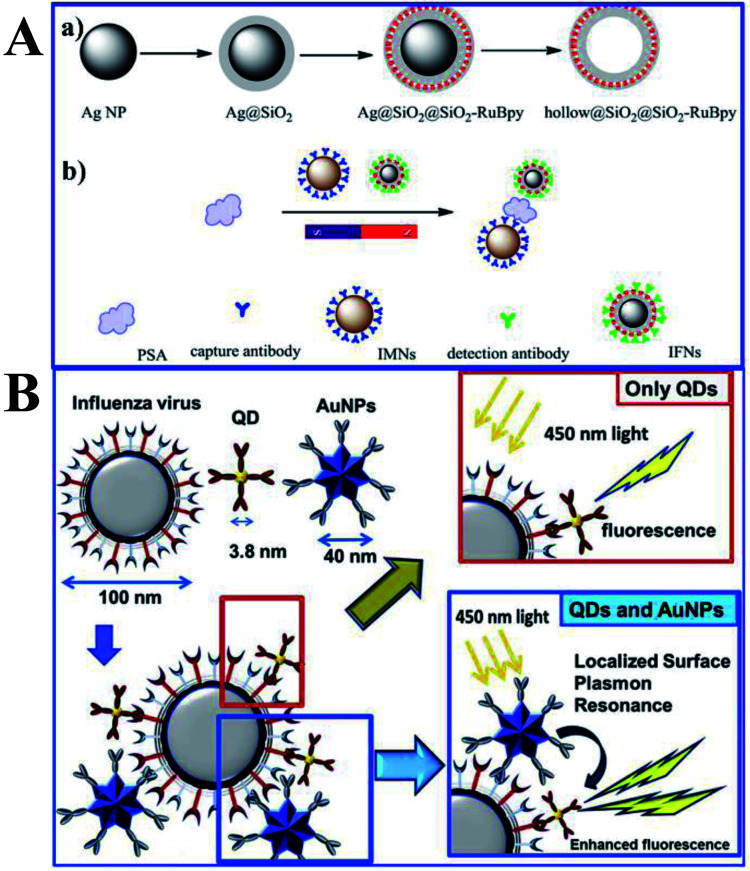
(A) Schematic showing synthesis processes of (a) Ag@SiO_2_@SiO_2_-RuBpy and hollow@SiO_2_@SiO_2_-RuBpy and (b) the sensing mechanism of PSA with the immunomagnetic nanospheres (IMNs) and immunofluorescent nanoparticles (IFNs), reprinted from ref. 159 with permission. (B) Schematic representations of the sensing principles of the MEF based fluorescence biosensor for the influenza virus. Reprinted from ref. 43 with permission.

As biochips based on plasmonic nanoparticles exhibit robust, rapid, and convenient detection, their fabrications have attracted increasing study. And the MEF phenomenon has already been widely exploited for immunoassays (as summarized in Table 4). For example, Xu and his co-workers developed a Ag@SiO_2_@SiO_2_-RuBpy core–shell composite, and a strategy for the detection of prostate specific antigen (PSA) by combining MEF and the magnetic separation technique was developed (as shown in Fig. 9A), which showed a good linear relationship between fluorescence intensity and the PSA concentration in the range of 0.1–100 ng mL^−1^.^159^ And it has also been successfully applied for the detection of PSA in human serum with high sensitivity and specificity, proving its potential in tumor diagnosis.

**Table 4:** MEF-based sensors used for the immunoassay

MEF sensors	Fluorophores used	Fluorescence enhancement factor	Target analytes	Relative merits (LOD refers to the limit of detection)
Ag@SiO_2_@SiO_2_-RuBpy^159^	RuBpy	3	PSA	LOD of 0.1–100 ng mL^−1^; MEF and magnetic separation was combined; potential application of tumor diagnosis
Alloyed quaternary CdSeTeS QDs and Au NPs^43^	CdSeTeS QDs	Not mentioned	Influenza virus	LOD of 10 PFU mL^−1^ for isolated H3N2
Size-encoded PMMB-based multiplexed suspension array^162^	Dyes	60	Multiple biomarkers	LOD of 100 fM; simultaneous detection of multiple targets with high output efficiency in complex samples; point-of-care detection
Ag NPs-gold nano film assembly^75^	Dyes	800	Antigen	Neglectable initial background signal; limitless potential applications in biosensing
Gold island films and CDs^38^	CDs	17.2	AFP	Dual amplification fluorescence assay; LOD of 94.3 fg mL^−1^; a wide linear detection range of 0.0005–5 ng mL^−1^
Patterned Au NPs^95^	FITC	Not mentioned	IgGs	LOD of 10 μg L^−1^; a linear response range of 10–100 μg L^−1^; direct detection of IgGs from patients’ urine without any pretreatments; point-of-care analysis
AgNCs^61^	Dye	4.6	CEA	LOD of 1 ng mL^−1^; incubation times were tremendously reduced by acoustic streaming with Rayleigh surface acoustic waves
Ag@SiO_2_@SiO_2_-RuBpy nanocomposites^28^	RuBpy	2.12	PSA	LOD of 0.20 ng mL^−1^; PSA can be detected in the range of 1–100 ng mL^−1^

Influenza viruses, which can cause flu infections, pose major threats to human health, and thus their specific and sensitive diagnosis is urgently needed. Takemura and his co-workers developed a solution-based nanobiosensor based on MEF of alloyed quaternary CdSeTeS QDs (QD) induced by Au NPs for the detection of influenza viruses.^43^ The QDs were conjugated with anti-hemagglutinin (HA) antibodies (anti-HA Abs), while AuNPs were conjugated with anti-neuraminidase (NA) antibodies (anti-NA Abs). With the presence of influenza viruses, antigen interactions occurred on their surface, which along with adjacent Au NPs triggered MEF of QDs, as shown in Fig. 9B. Detection limits of 0.03 and 0.4 pg mL^−1^ were obtained for influenza H1N1 viruses in deionized water and human serum respectively. And the detection of the clinically isolated H3N2 was also accomplished with a detection limit of 10 PFU mL^−1^.

As there may be more than one definitive biomarker that can reliably diagnose early stages of many diseases, simultaneous assays with high specificity and sensitivity are necessary.^128^ Moreover, driven by demands for cost efficiency, there are also increasing needs to acquire more information from a single experiment. Multiplexed assays can be used to measure multiple target analytes in a single run of the assay, which includes protein and nucleic acid-based multiplexing.^161,162^

**Fig. 10:**
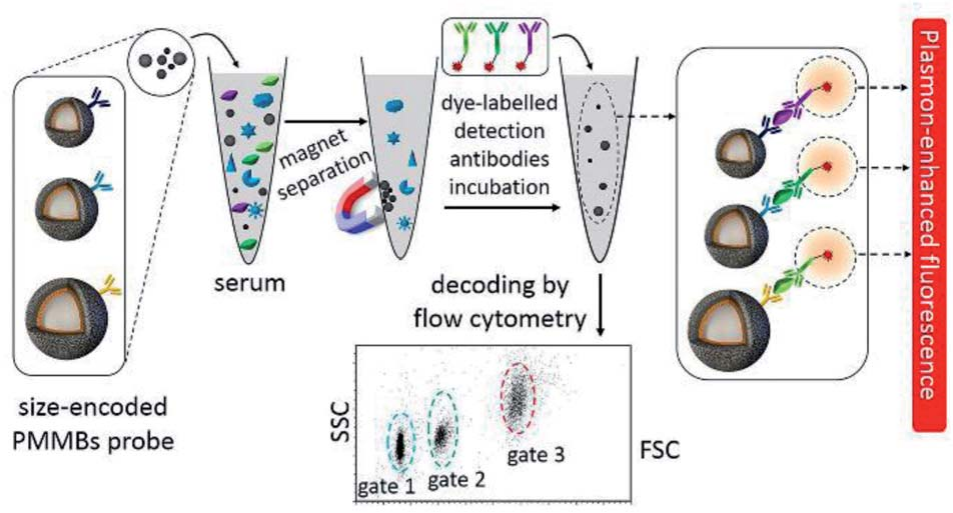
Schematic showing the experimental design: PMMBs of different sizes were conjugated with different capture antibodies for simultaneous detection of multiple targets with great sensitivity *via* MEF. Reprinted Scheme 1 with permission from Chao Yuan, Yunte Deng, Xuemeng Li, Chengfei Li, Zhidong Xiao and Zhuang Liu, *Anal. Chem.*, 2018, **90**, 8178–8187 (ref. 160). Copyright 2018 American Chemical Society.

Considering the perfect specificity of immunoassays, multiplex detection of different biomarkers can be realized by conjugating plasmonic NPs with different capture antibodies. Yuan and his co-workers reported a kind of size-encoded plasmonic magnetic microbead (PMMB)-based multiplexed suspension array for simultaneous detection of multiple biomarkers, see Fig. 10. These PMMBs realized 60-fold fluorescence enhancement, and have improved the detection limit by 2-orders of magnitude toward 100 fM for biomarkers.^160^ The multiplexing ability of the as-prepared PMMB platform is particularly attractive for simultaneous detection of multiple targets with high output efficiency in complex samples. And we predict that this MEF-based PMMB could be employed as the next generation probe for biomarker detection and disease diagnosis in a multiplexed manner.

**Fig. 11:**
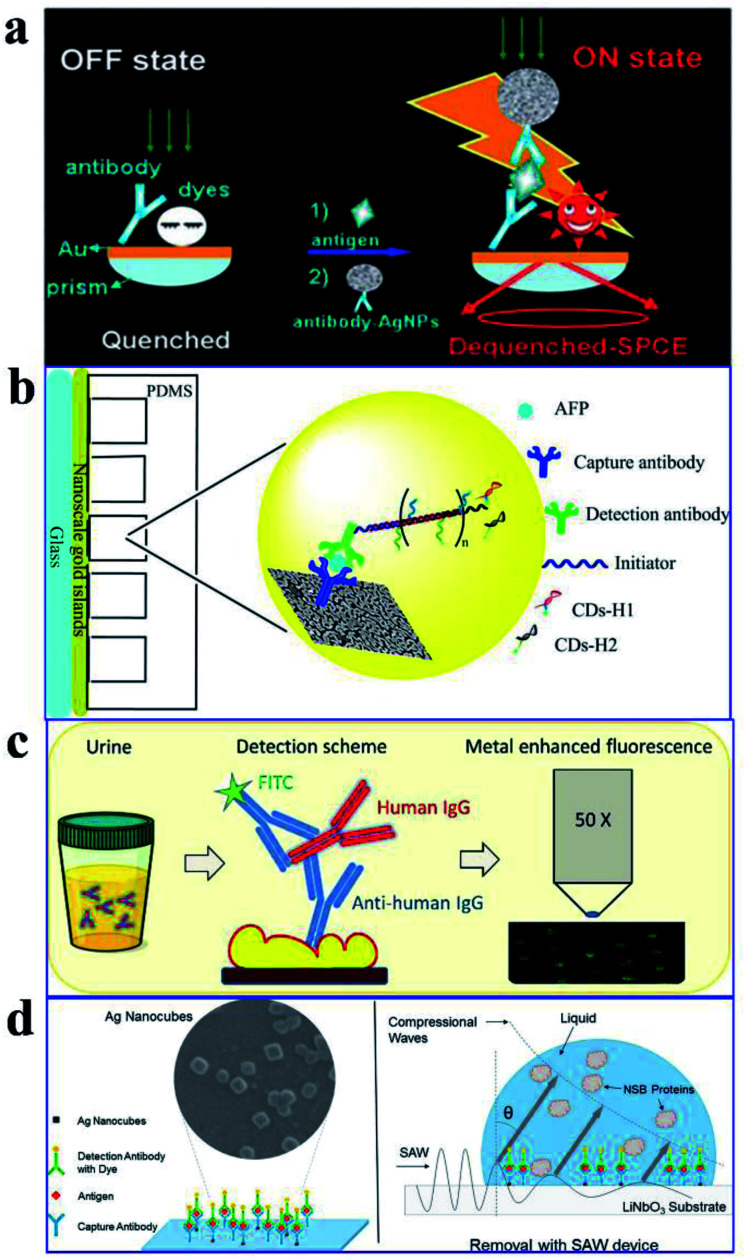
(a) Schematic illustration of the as-assembled sandwich structure for the immunoassay *via* dequenching. Reproduced from ref. 75 with permission from the Royal Society of Chemistry. (b) Schematic detection mechanism of AFP with the immuno-HCR and CD MEF. Reprinted TOC figure with permission from Dang-Dang Xu, Cui Liu, Cheng-Yu Li, Chong-Yang Song, Ya-Feng Kang, Chu-Bo Qi, Yi Lin, Dai-Wen Pang and Hong-Wu Tang, *ACS Appl. Mater. Interfaces* 2017, **9**, 37606–37614 (ref. 38). Copyright 2017 American Chemical Society. (c) Schematic illustration of the detection mechanism for IgGs in urine: antibodies tethered to the Au NP substrate to recognize the antigen (human IgG), then secondary antibodies tagged by FITC bound to the human IgG in a sandwich configuration for fluorescence imaging measurements. Reprinted TOC figure with permission from Bartolomeo Della Ventura, Monica Gelzo, Edmondo Battista, Alessandro Alabastri, Andrea Schirato, Giuseppe Castaldo, Gaetano Corso, Francesco Gentile and Raffaele Velotta, *ACS Appl. Mater. Interfaces*, 2019, **11**, 3753–3762 (ref. 95). Copyright 2019 American Chemical Society. (d) Schematic showing (left) the sensing mechanism for CEA using the Ag nanocube-based plasmon substrate, and (right) removal of nonspecifically bound (NSB) proteins with SAW devices. Reprinted TOC figure with permission from Jun Liu, Shuangming Li and Venkat R. Bhethanabotla, *ACS Sens.*, 2018, **3**, 222–229 (ref. 61). Copyright 2018 American Chemical Society.

As plasmon nanostructures can effectively enhance emission, the fluorescence can be modulated *via* assembly of various plasmon structures.^75^ Using the Ag NP-gold nano film assembly *via* biorecognition binding, an over 800 fold dequenched fluorescence signal was observed by Cao and his co-workers.^75^ As shown in Fig. 11a, the sensing of target antigens can be conveniently confirmed by the intense fluorescence signal. As this Ag NP-gold nano film assembly works in an ‘off–on’ mode with a neglectable initial background signal, it may have limitless potential applications in biosensing. Their study may pave ways for plasmonic coupling assembly and application.

By incorporating immune hybridization chain reaction (HCR) and CD MEF, Xu and his co-workers constructed a dual amplification fluorescence sensor for alpha fetal protein (AFP).^38^ With the help of the capture-antibody coated plasmonic slide of gold island films (Fig. 11b) and the detection antibody-conjugated oligonucleotide initiator, HCR was triggered after the introduction of CD-tagged DNA hairpins, which are complemented to the oligonucleotide initiator. In this cleverly designed AFP sensor, CD emission was greatly enhanced by the gold nano island film, HCR provided secondary fluorescence amplification simultaneously, and the two together resulted in a 17.2-fold total signal amplification. A wide linear detection range of over 5 orders of magnitude between CD emission and AFP concentration (0.0005–5 ng mL^−1^) was obtained, with a low detection limit of 94.3 fg mL^−1^. They also realized detection of real samples using this dual amplification fluorescence assay method.^38^

An immunosensor for immunoglobulins (IgGs), with the fluorescence signal enhanced by patterned Au NPs and specificity realized *via* biological functionalization (see Fig. 11c), was developed.^95^ The as-prepared device can directly detect IgGs from a drop of a patient's urine without any pretreatments, and a detection limit of 10 μg L^−1^ was realized with a linear response range of 10–100 μg L^−1^. Further experiments proved its excellent specificity rarely interfered by other biomolecules and reliable analysis results comparable with those obtained using standard techniques.

Liu and his co-workers developed an immunofluorescence probe by combining MEF with the surface acoustic wave (SAW) technique to lower the detection limit for the carcinoembryonic antigen (CEA),^61^ which is a prognostic biomarker of colorectal cancer (see Fig. 11d). By incubating with 50 nm silver nanocubes (AgNCs) on a SAW device with an optimal surface density, emission was plasmon enhanced, which improved sensitivity to antigens by a factor of 6 and lowered the detection limit down to below 1 ng mL^−1^. Moreover, nonspecifically bound proteins were much more effectively removed and incubation times was tremendously reduced by the introduction of acoustic streaming. Overall, clinical levels of colorectal cancer biomarkers can be detected using this AgNC-based MEF probe.

Deng and his co-workers constructed another core–shell Ag@SiO_2_@SiO_2_-RuBpy nanocomposite for the detection of prostate specific antigen (PSA) using the target-triggered MEF ‘turn-on’ strategy, with RuBpy acting as the donor and BHQ-2 as the acceptor. BHQ-2 was brought to close proximity to the surface of the RuBpy-doped silica shell after hybridization occurred (‘off’ state). However, with the addition of target PSA, the BHQ-PSA aptamer could be dissociated from the RuBpy-doped silica shell (‘on’ state). Thus PSA quantitative analysis can be realized by recording fluorescence intensity, and a detection limit of 0.20 ng mL^−1^ was obtained under current experimental conditions.^28^

### 3.2 RNA and DNA detection

MicroRNAs (miRNAs), as noncoding RNAs, play crucial roles in regulating diverse physiological functions. Expression levels of tumor-related miRNAs may predict tumor growth and invasiveness, and thus their accurate detection is important. However, as a result of their low expression levels, miRNA detection remains a challenge. And MEF has also been applied for RNA and DNA analysis for its much lower detection limit (see Table 5).

**Table 5:** MEF-based sensors for RNA and DNA

MEF sensors	Fluorophores used	Target analytes	Relative merits (LOD refers to the limit of detection)
Au NRs^98^	Cy5	miRNA	LOD of 97.2 × 10^−18^ M; dual-amplification of the fluorescence signal was realized *via* combining MEF and a strand displacement amplification (SDA) reaction
Ag film/Ag zigzag nanorod array multilayer film	alex488	DNA	LOD of 0.01 pm
Au NPs^118^	QDs	DNA	LOD of 19.6 pg
GNR array^100^	Fluorophore	ssDNA	A linear range from 10 pM to 10 nM
ORA-enabled molecular beacons^152^	CDs	DNA	LOD of 300 fM; with four orders of sensitivity improvement

**Fig. 12:**
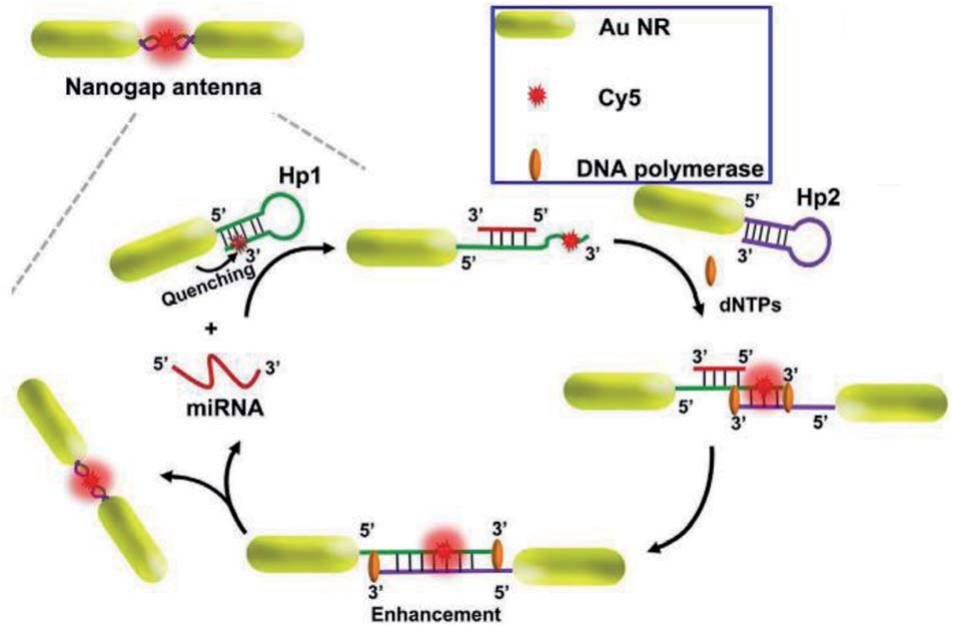
Schematic showing the assembly of nanogap antennas and sensing mechanisms for miRNA detection. Reprinted from ref. 98 with permission.

As reported, a stronger electromagnetic field can be generated in the gap regions of two neighboring particles, which will result in a much more dramatic fluorescence enhancement.^163^ Thus a gold nanogap antenna through target-triggered assembly of Au NRs was constructed by Peng and her co-workers. Dual-amplification of the fluorescence signal was realized *via* combining the MEF effect of nanogap antennas and a strand displacement amplification (SDA) reaction, see Fig. 12. In the presence of target miRNA, fluorophores can be settled into the gap region of the gradually formed end-to-end Au NR dimers. Thus quenched fluorescence induced by the Au NRs exhibited a dramatic enhancement by the MEF of the nanogap antennas, and a ‘turn on’ fluorescence signal was observed. The SDA reaction resulted in secondary fluorescence amplification simultaneously. A low detection limit of 97.2 × 10^−18^ M miRNA was realized by combining this method with the single-molecule counting technique.^98^ Moreover, this proposed method has potential in monitoring expression levels for low-abundance nucleic acid biomarkers *via* miRNA imaging.

**Fig. 13:**
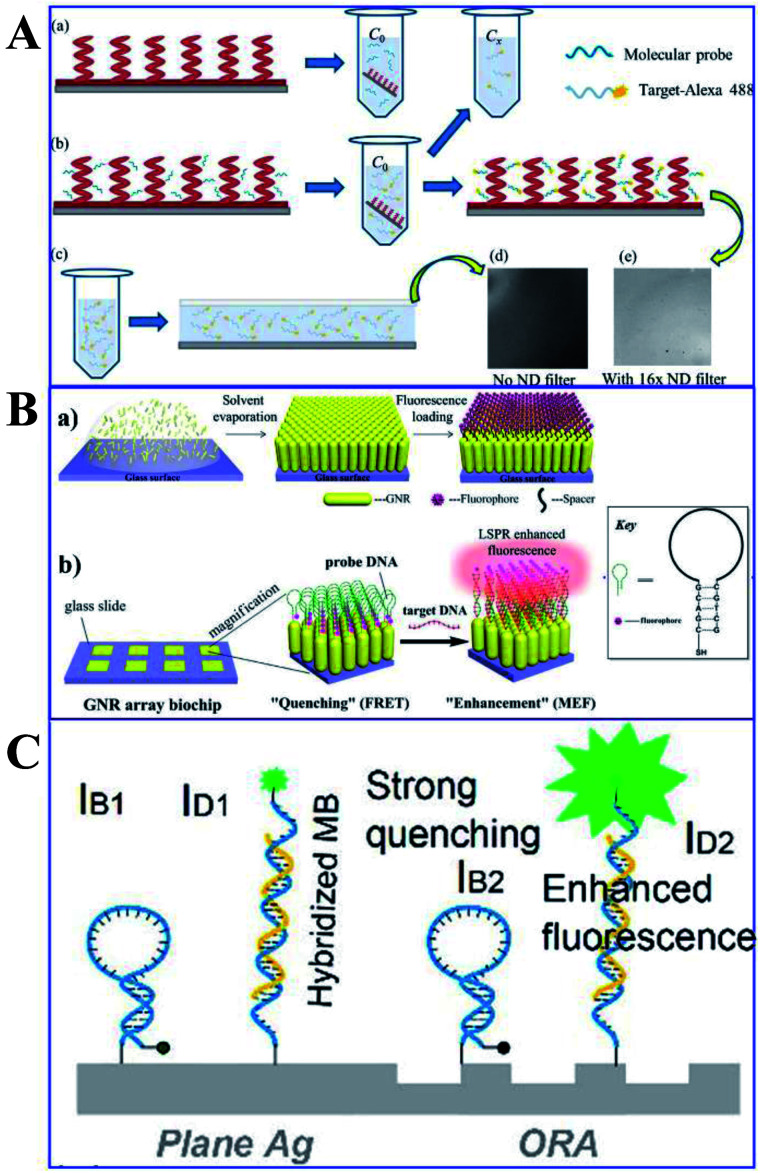
(A) Schematic of the (a) molecular probe binding to AgZNRs and (b) fluorophore labelled target oligonucleotide hybridizing with the molecular probe, with its fluorescence image in (e). (c) A drop of the target-alexa488 is sandwiched between the Si wafer and a piece of cover slip for fluorescence measurement, with the fluorescence image shown in (d). Reprinted from ref. 118 with permission. (B) Schematic showing (a) the fabrication processes of the ordered standing GNR, and (b) practical applications of the GNR array chip for DNA detection. Reprinted TOC figure with permission from Zhong Mei and Liang Tang, *Anal. Chem.*, 2017, **89**, 633–639 (ref. 100). Copyright 2017 American Chemical Society. (C) Schematic showing MBs anchored on the ORAs and plane silver surfaces respectively. IB1/IB2: quenched background intensities on plane silver/ORAs, ID1/ID2: fluorescence signals of hybridized MBs on plane silver/ORA surfaces, respectively. Reprinted TOC figure with permission from Akash Kannegulla, Ye Liu, Bo Wu and Li-Jing Cheng, *J. Phys. Chem. C*, 2018, **122**, 770–776 (ref. 152). Copyright 2018 American Chemical Society.

Protein and DNA detections are important steps for biological and diagnostic assays. Thus Ji and her co-workers developed a Ag film/Ag zigzag nanorod array multilayer film for DNA detection (as shown in Fig. 13A). With a folding number of 7, a 14-fold fluorescence enhancement of alex488 was obtained, and the detection limit was determined to be 0.01 pm. Moreover, another study by Li and his co-workers using Au NP-enhanced QD emission for detection of DNA was developed, and a high sensitivity of 19.6 pg was established by controlling the distance between the QDs and Au NPs using different numbers of oligonucleotides.^118^ Mei and his co-workers developed an innovative gold nanorod (GNR) array biochip with vertical standing arrays of ordered GNR on a glass surface, as shown in Fig. 13B.^100^ As shown in Fig. 13B, the initial fluorescence of the biochip is minimal when the hairpin-structured ssDNA probe is conjugated to the array, as a result of quenching induced by nearby GNRs. However, the hairpin loop could be opened up with the formation of duplex DNA *via* hybridization, which brought the fluorophore away from the GNR array (45-nucleotide-long). And this process can induce a dramatically intensified fluorescence signal, which could be used for quantitative ssDNA detection with a linear range from 10 pM to 10 nM. Further, Kannegulla and his co-workers reported an Ag open-ring nanoarray (ORA)-enabled molecular beacon (MB) probe for DNA detection (see Fig. 13C), which yielded a detection limit of ∼300 fM (equivalent subattomoles), four orders of improvement compared with that using a plane Ag substrate.^152^ The ultrahigh sensitivity resulted from both the intensified fluorescence signal due to MEF of the ORA-enabled MB platform and the reduced background signal level.

### 3.3 Enzyme detection

Acetylcholinesterase (AChE) in human blood is considered to be a biomarker of neurotoxin exposure, which inhibits AChE. Thus its trace detection in human blood is important. So far, scientists have also developed various MEF-based sensors for enzyme detection (see Table 6). For example, Ma and his co-workers designed a new kind of Ag@SiO_2_ core–shell NP based *in situ* probe for AChE by combining the AChE catalytic reaction with MEF.^94^ The surface of Ag@SiO_2_ NPs is negatively charged, while AChE can catalyze acetylthiocholine (ATCh) hydrolysis to positively charged thiocholine (TCh), and thus the negatively surface charge of the core–shell Ag@SiO_2_ NPs can be reversed as a result of electrostatic adsorption of TCh. Then the negatively charged fluorescent dye (8-hydroxypyrene-1,3,6-trisulfonic acid, HPTS) was confined to the Ag@SiO_2_ NP surface, which resulted in an enhanced fluorescence signal. A dynamic range of 0–0.005 U mL^−1^ AChE was detected *via* this mechanism, with a detection limit of 0.05 mU mL^−1^.^94^ This work provides a new way of design for MEF-based probes for biomolecules.

**Table 6:** MEF-based sensors for enzymes

MEF sensors	Fluorophores used	Target analytes	Relative merits (LOD refers to the limit of detection)
Ag@SiO2 NPs^94^	HPTS	ATCh	Combining the AChE catalytic reaction with MEF; a detection range of 0–0.005 U mL^−1^; LOD of 0.05 mU mL^−1^
Au NBPs^92^	Cy5.5	Telomerase	LOD of 23 HeLa cells with a dynamic range of 40–1200; potential in clinical diagnosis
Nano-silvered 96-well plate^96^	FITC	Trypsin	LOD of 1.89 ng; however, the present work needs lengthy incubation times and washing steps

**Fig. 14:**
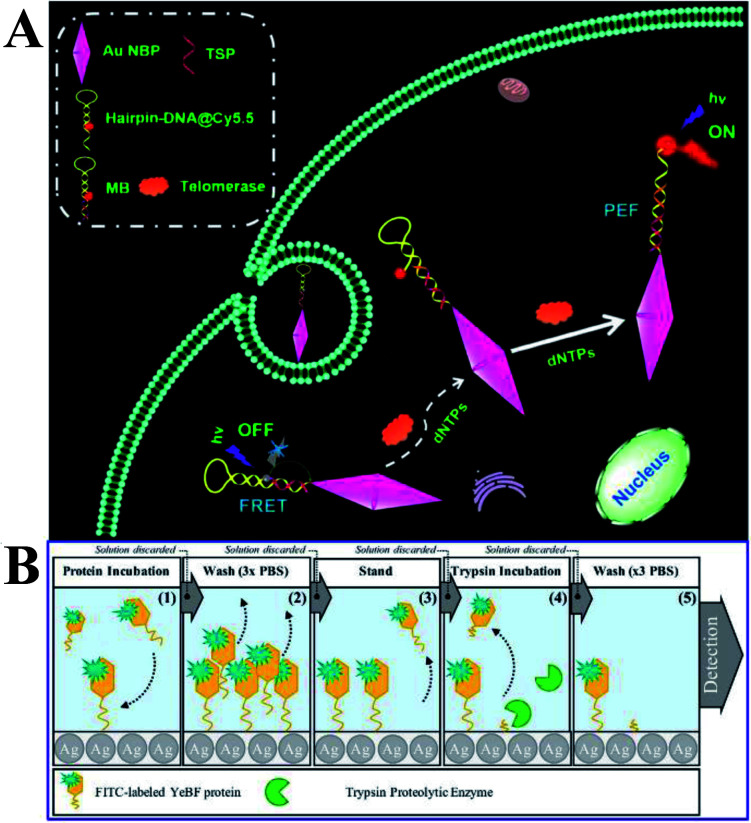
(A) Schematic showing the MEF-based telomerase nanoprobe, which can realize *in situ* analysis of telomerase activity in live cells. Reprinted TOC figure with permission from Shenghao Xu, Liping Jiang, Yongyin Nie, Jun Wang, Haiming Li, Yuanyuan Liu, Wei Wang, Guiyun Xu and Xiliang Luo, *ACS Appl. Mater. Interfaces*, 2018, **10**, 26851–26858 (ref. 92). Copyright 2018 American Chemical Society. (B) Schematic showing experimental procedures and assay methods. Reprinted from ref. 96 with permission.

Moreover, Xu and his co-workers developed a novel solution-based nanoprobe for *in situ* fluorescence visualized ‘turn on’ detection of telomerase in live cells,^92^ which is one of the most common biomarkers for cancer diagnosis and pathogenesis. In this work, gold nanobipyramids (Au NBPs) were used as both the fluorescence resonance energy-transfer (FRET) quencher and MEF signal enhancement substrates of Cy5.5. After being conjugated to the Au NBPs, as a result of FRET, the fluorescence of Cy5.5 was totally quenched, while with the addition of deoxyribonucleotide triphosphates (dNTPs) and telomerase, the hairpin loop was opened, followed by the dequenching of Cy5.5. The best MEF factor can be obtained by adjusting the number of oligonucleotide bases. Thus this kind of nicked molecular beacon-functionalized Au NBP is a dual-functional substrate, and a low detection limit of 23 HeLa cells with a dynamic range of 40–1200 was obtained (Fig. 14A). *In situ* dynamic telomerase activity fluorescence imaging in live HeLa cells was realized as a result of its outstanding biocompatibility, stability and specificity. And cancer cells were also successfully distinguished from co-cultured normal ones, proving its potential in clinical diagnosis.^92^ This work undoubtedly demonstrated a new pathway for designing sensitive and specific MEF based probes for cancer-related biomolecules.

Proteases are recognized as biomarkers of various diseases. Lucas and his co-workers reported a MEF-based ‘turn-off’ assay for proteolytic enzymes using a nano-silvered 96-well plate. And the use of fluorescein isothiocyanate-labeled YebF protein as a coating layer for the enzymatic activity assay using trypsin as the model enzyme was demonstrated (as shown in Fig. 14B).^96^ A detection limit of 1.89 ng was achieved, corresponding to 10% fluorescence quenching. However, the present work needed lengthy incubation times and washing steps, and they also put forward that the overall assay time might be shortened *via* a microwave-accelerated MEF technology in the future assay design.

### 3.4 ATP detection

Adenosine triphosphate (ATP), as a typical energy storage molecule, can transport chemical energy for cellular metabolism processes. It is an important biomarker for disease diagnosis. Compared to electrochemical and colorimetric methods, fluorescent sensors exhibit the ability to measure ATP in real-time. However, lower detection limits of the present fluorescence methods for ATP are still at ranges of millimolar to micromolar. Developing ATP fluorescent sensors with high sensitivity and stability is highly desirable, which can be realized by combining MEF with fluorescence methods (as summarized in Table 7). And we will review these advances in this part.

**Table 7:** MEF-based sensors for ATP

MEF sensors	Fluorophores used	Target analytes	Relative merits (LOD refers to the limit of detection)
Ag@SiO_2_ NPs core–shell structure^120^	PG	ATP	LOD of 14.2 nM; a wide detection range
Gold nanorods^164^	IR dye 800CW	ATP	LOD of 10 and 0.634 nM; successfully applied to detect ATP in rat brain
Silver island films (SIFs)^151^	PG	ATP and thrombin	LOD of 1.3 and 0.073 nM, and linear detection ranges from 10 nM to 100 μM and 0.1 nM to 100 nM in the logarithmic scale for ATP and thrombin respectively

**Fig. 15:**
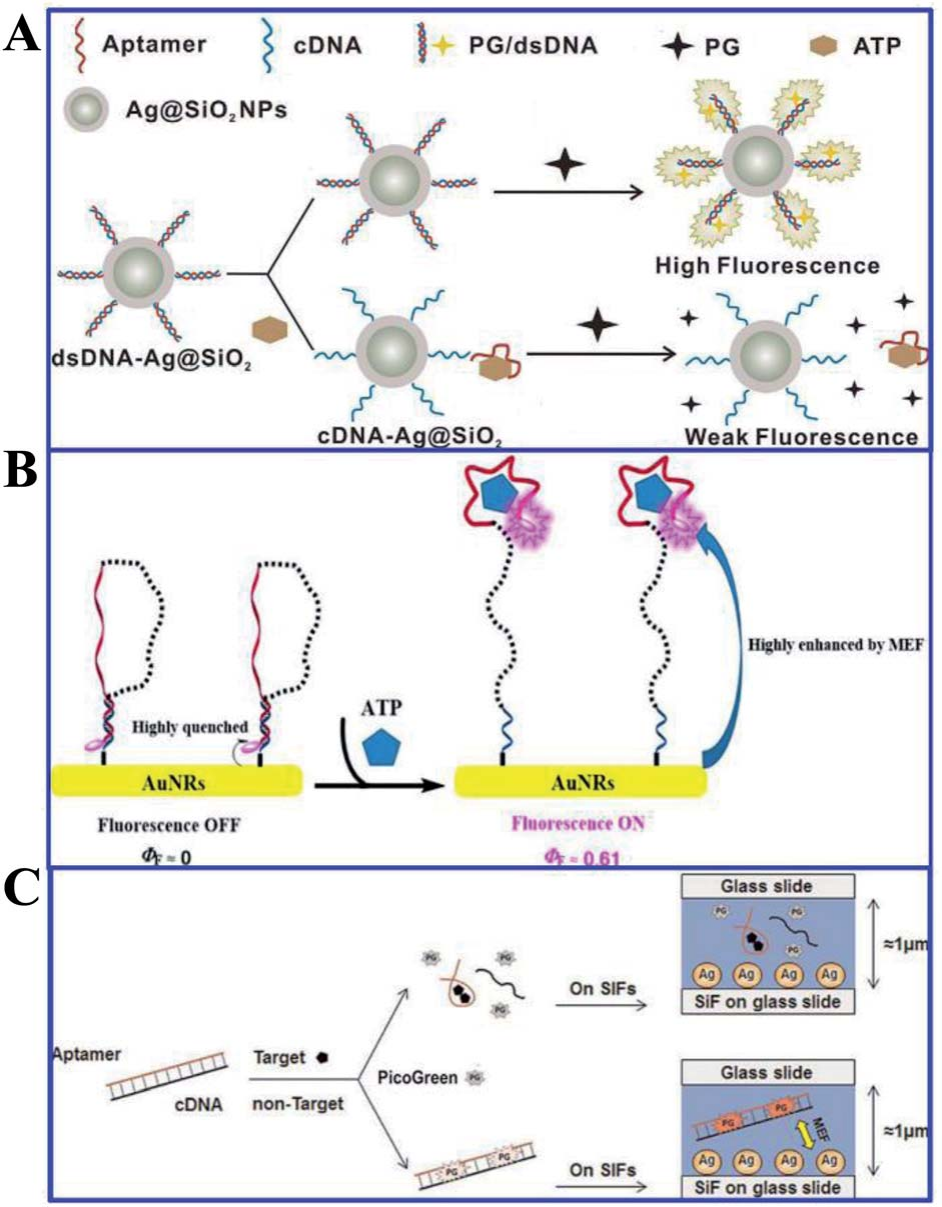
(A) Schematic illustration of the sensing procedure for ATP detection. Reprinted from ref. 120 with permission. (B) Schematic illustration of the MEF-based NIR fluorescence detection of ATP. Reprinted TOC figure with permission from Manli Yu, Yishan Yao, Bo Cui, Changjiao Sun, Xiang Zhao, Yan Wang, Guoqiang Liu, Haixin Cui and Zhanghua Zeng, *ACS Appl. Nano Mater.*, 2019, **2**, 48–57 (ref. 164). Copyright 2019 American Chemical Society. (C) Schematic illustration of the universal and label-free sensing mechanisms *via* target-induced aptamer conformational changes and MEF effects of SIFs. Reprinted from ref. 151 with permission.

Song and his co-workers presented a label-free aptasensor by using the Ag@SiO_2_ NPs core–shell structure and the common nucleic acid stain PicoGreen (PG) as a fluorescent indicator, for highly sensitive and selective detection of ATP in aqueous solutions (see Fig. 15A).^120^ A significant fluorescence reduction was observed in the presence of ATP, as a result of the aptamer release from the complementary DNA (cDNA)/aptamer duplexes confined on the Ag@SiO_2_ NP surface.^120^ And this aptasensor achieved a wide linear detection range for ATP with a detection limit of 14.2 nM, exhibiting its excellent assay performances. This work may provide new avenues for assembly of MEF-based label-free biosensors.

**Table 8:** MEF-based sensors for other biomolecules

MEF sensors	Fluorophores used	Target analytes	Relative merits (LOD refers to the limit of detection)
Au@SiO_2_ based core–shell nanostructures^56^	TCPP	PPi	LOD of 8.20 × 10^−7^ M; cell imaging
Au NBPs + SiO_2_ (ref. 62)	NIR dyes	PPi and microRNA	LOD of 80 nM and 8.4 pM respectively
ZnSa NW–Ag NP 1D hybrid nanostructure^51^	DA	DA	LOD of 3 nM; excellent selectivity and long-term stability
Molecularly imprinted core–shell Ag@SiO_2_ NPs^99^	RF	RF	Molecular imprinting combined with MEF
Au@SiO_2_ NPs^106^	BSNVA	PrPSc	LOD of 10 pM; monitoring protein conformational conversion in human serum samples

Near-infrared fluorescence (NIR) is extensively used in biological fields for its high penetration, low photothermal damage and immunity from autofluorescence. However, common NIR fluorophores suffer from low quantum efficiency. As optical antennas with anisotropic nanostructures have been widely used in the field of MEF, Yu and her co-workers developed a NIR based MEF platform of functionalized gold nanorods for the bioassay of ATP.^164^ As shown in Fig. 15B, in the absence of ATP, the self-hybridized DNA sequence brought the IR dye 800CW close to the AuNR surface, which resulted in a ‘fluorescence off’ state, while the presence of ATP could disrupt this self-hybridization and push IR dye 800CW away from the AuNR surface, as a result of its higher specific affinity with the aptamer moiety. A low detection limit of 0.634 nM was obtained for ATP as a result of the greatly enhanced quantum efficiency of IR dye 800CW from 0 to 0.61. The biosensor was also successfully applied to detect low levels of ATP in rat brain, demonstrating its applicability for monitoring intracellular ATP.

Aptamers are a kind of recognition molecule composed of single-stranded nucleic acid that folds into special 3-D structures, and can specifically bind to targets with high affinity.^165^ They have advantages of low molecular weight, high stability and ease of chemical synthesis compared to antibodies. Thus Song and his co-workers developed a label-free fluorescence aptasensor for highly sensitive and selective detection of ATP and thrombin, with PicoGreen (PG) used as a signal molecule and silver island films (SIFs) as MEF substrates for signal enhancers.^120^ PG fluorescence can be magnified by SIFs without ATP or thrombin, as shown in Fig. 15C. However, with the presence of ATP or thrombin, as the aptamers underwent structure switching, PG fluorescence intensity was reduced. And detection limits of 1.3 nM and 0.073 nM were obtained for ATP and thrombin respectively using this method. ATP and thrombin could be linearly detected in ranges from 10 nM to 100 μM and 0.1 nM to 100 nM in the logarithmic scale, respectively. And this aptamer can also be reliably used for ATP measurements in biological samples.

### 3.5 Detection of other biomolecules

Besides applications in immunoassays, RNA/DNA, enzyme and ATP detection, fluorescence detection of other biomolecules such as PPi, dopamine (DA) and riboflavin (RF) using MEF has rapidly developed, and we will summarize these recent reports in this section (Table 8).

**Fig. 16:**
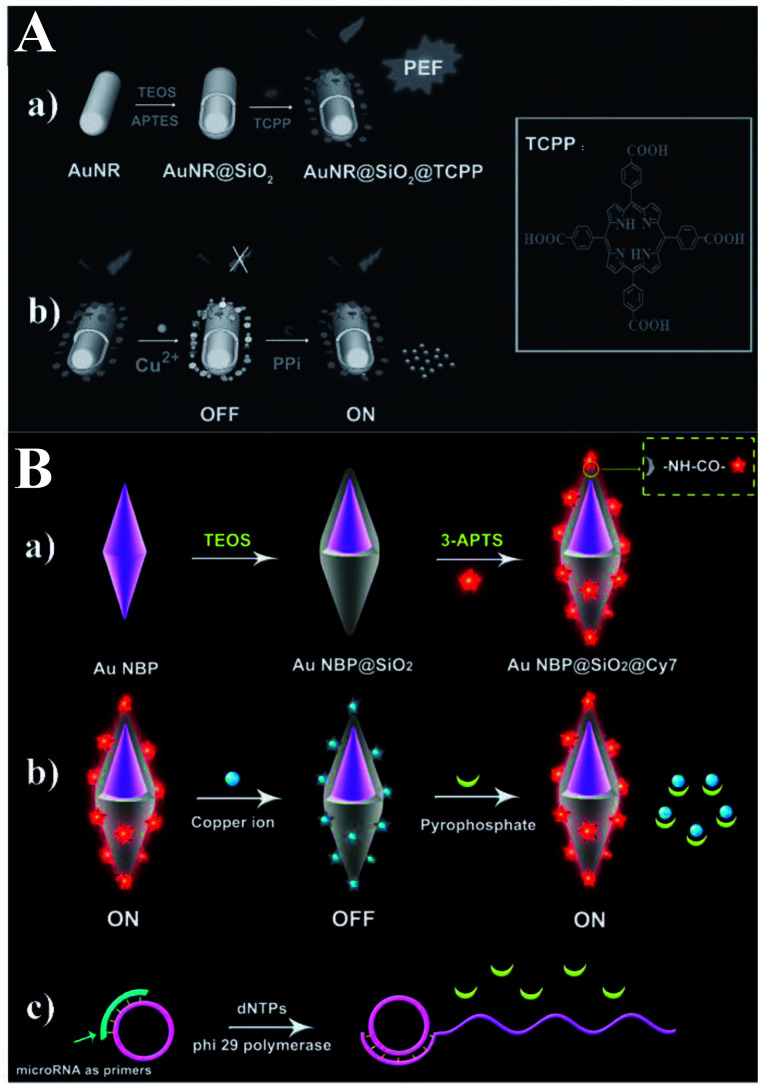
(A) Schematic showing the (a) preparation processes of PEF-based core–shell AuNR@SiO_2_@TCPP NPs, and (b) the sensing mechanism of the MEF-based core–shell AuNR@SiO_2_@TCPP NPs for PPi. Reprinted from ref. 60 with permission. (B) Schematic showing (a) the synthesis processes of Au NBP@SiO_2_@Cy7 NPs and the detection mechanisms for (b) PPi and (c) microRNA. Reprinted Scheme 1 with permission from Caixia Niu, Quanwei Song, Gen He, Na Na and Jin Ouyang, *Anal. Chem.*, 2016, **88**, 11062–11069 (ref. 62). Copyright 2016 American Chemical Society.

Based on a similar sensing mechanism for Cu^2+^, PPi and PPase previously referred to in ref. 56, another two Au@SiO_2_ based core–shell nanostructures composed of Au nanorods (NRs) and elongated gold nanobipyramids (Au NBPs) encapsulated by SiO_2_ were reported and used for NIR detection of PPi,^60,62^ which is closely related to the DNA replication process and the genetic information expression. As shown in Fig. 16A, meso-tetra(4-carboxyphenyl)porphyrin (TCPP) molecules were covalently immobilized onto the outer shell surface of the AuNR@SiO_2_.^60^ As a result of the strong affinity between Cu^2+^ and PPi, the turn-off state of TCPP-Cu^2+^ can be disassembled, and fluorescence was recovered. Combined with the MEF imparted by AuNRs, a detection limit of 8.20 × 10^−7^ M PPi was realized. Cell imaging using this sensor was also realized, proving its potential applications in biological mechanism studies.

Both experiments and theoretical simulations indicate that Au NBPs can induce signal enhancement several times higher than that of Au NRs with similar longitudinal plasmon resonance wavelength. Thus Niu and her co-workers demonstrated a novel NIR MEF system composed of an elongated gold nanobipyramid (Au NBP) antenna core, a silica shell and a NIR dye (see Fig. 16B).^62^ The largely enhanced fluorescence could be quenched by Cu^2+^ and further recovered by PPi, owing to the stronger affinity between Cu^2+^ and PPi. And this provided a method for ‘switch-on’ detection of PPi with a limit of 80 nM in aqueous solutions.

Furthermore, the probe was used for microRNA detection with a low detection limit of 8.4 pM.^62^

**Fig. 17:**
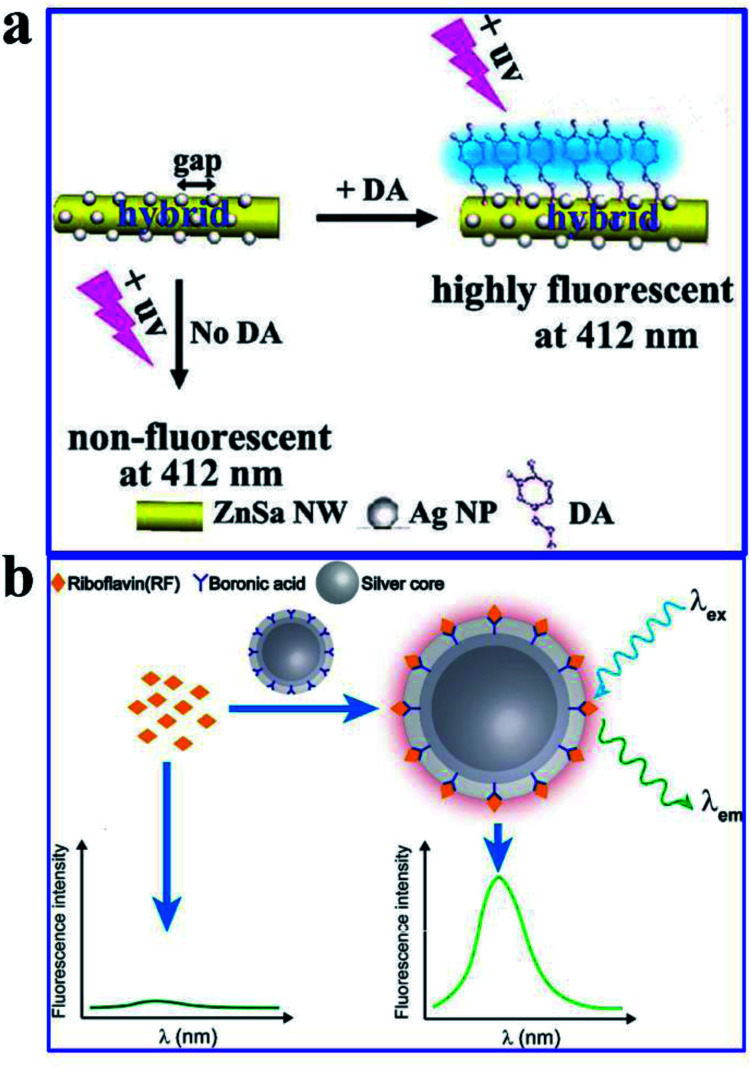
(a) Schematic illustration of the hybrid that can improve DA fluorescence detection, *λ*_em_ = 350 nm. Reproduced from ref. 51 with permission from the Royal Society of Chemistry. (b) Schematic showing the preparation processes and sensing mechanisms of the molecularly imprinted core–shell Ag@SiO_2_ NPs for the fluorescence assay. Reprinted from ref. 99 with permission.

Dopamine (DA) acting as an important neurotransmitter has always been a bioresearch focus. Yang and his co-workers fabricated a novel zinc–salophen (ZnSa) complex nanowire (NW)–Ag NP 1D hybrid nanostructure. Narrow gaps between the Ag NPs, acting as optical antennas that can produce a largely enhanced electrical field for signal amplification, are shown in Fig. 17a. Thus sensing occurring at these nanogaps can realize improved performance. The specific binding of DA with ZnSa NWs realized DA selective detection, while the introduction of Ag NPs induced a substantially improved performance for DA detection *via* remarkable fluorescence enhancements. And a detection limit as low as 3 nM was obtained. Excellent selectivity and long-term stability of the hybrid nanostructure were also predicted in the article.^51^

Molecularly imprinted polymers (MIPs) are a kind of biomimetic receptor, synthesized through polymerization reactions in the presence of a template molecule. They are tailor-made for target molecules, and have antibody-like specific binding properties.^166^ Herein, He and his co-workers reported a molecularly imprinted core–shell Ag@SiO_2_ NP for sensitive and specific MEF assay of riboflavin (RF),^99^ as shown in Fig. 17b. Their work might pave the way for sensitive and specific MEF assays based on molecularly imprinted plasmonic nanostructures.

**Fig. 18:**
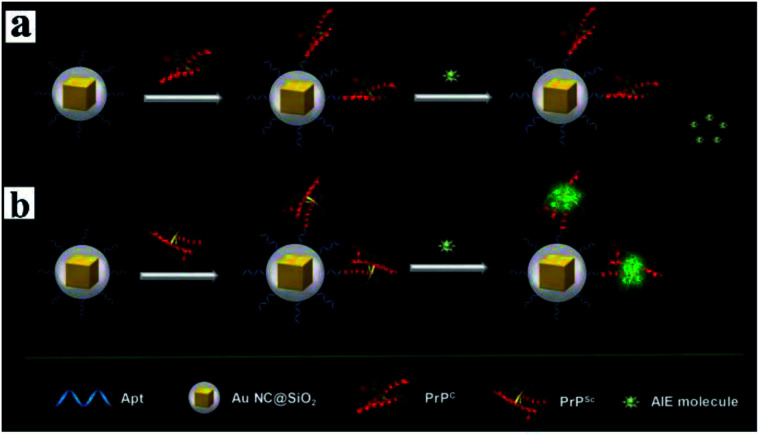
Schematic illustration of the MEF sensor based on AIE molecules for the detection of (a) PrPC and (b) PrPSc. Reprinted from ref. 106 with permission.

Aggregation-induced emission (AIE) molecules, which are non-emissive in the dissolved state while highly fluorescent in the aggregated state, have advantages of a large Stokes' shift, excellent photostability, and high signal-to-noise ratio.^167^ Thus Cui and her co-workers developed a MEF sensor based on AIE molecules for monitoring protein conformational changes.^106^ A water-miscible sulfonate salt of 9,10-bis(2-(6-sulfonaphthalen-2-yl)vinyl)anthracene (BSNVA) with excellent AIE properties was introduced into the MEF system to prepare the MEF-AIE sensor, using a PrP aptamer as the bridge (as schematically shown in Fig. 18). When mixed with cellular prion protein (PrPc), the MEF-AIE sensor is almost non-emissive, while brightly fluorescent when mixed with disease-associated prion protein (PrPSc). Thus protein conformational conversion can be monitored through this PEF–AIE sensor. And a detection limit of 10 pM lower than that of the traditional AIE probe has been achieved in human serum samples.^106^ This work realized signal amplification without labeling, and also provided new insights for protein detection and conformational monitoring.

## 4 Conclusions and prospects

In this review, we summarized the recent key advances of MEF-based fluorescence sensing, including design, synthesis, assembly and applications of both solution-based colloidal NPs and plasmonic chip platforms. So far, applications of MEF-based fluorescence sensing are at varying degrees of progress. In general, both environmental detection and bioanalysis of special target analytes using plasmonic MEF have been extensively studied, and much lower detection limits and more reliable results have been achieved. Future work may focus on improving the selectivity and anti-interference performance of sensors, which could be realized by combining MEF-based fluorescence with other techniques including SERS, colorimetric methods, molecular imprinting and even immunoassays or aptasensor techniques. In particular, especially for bioassays, as there are usually more than one biomarker that can diagnose early stages of special diseases, we forecast that multiplexed assays using protein and nucleic acid could be a promising development direction. Furthermore, by preparing heteronanocomposite plasmonic nanostructures, owing to the synergistically enhanced optical properties of individual components and new features arising from the integrated systems, unexpected or improved sensing performances may be realized.^168^ Lastly, integration of plasmonic MEF fluorescence sensing with portable platforms, such as wearable devices and microfluidics has always been the goal of current research, which would greatly accelerate transformations from conventional benchtop assays to powerful point-of-care tests. However, challenges still remain for its implementation, and we expect that this goal can be achieved in the near future.

## Conflicts of interest

There are no conflicts to declare.

## Supplementary Material
